# Supplementary material for: Machine learning-informed and synthetic biology-enabled semi-continuous algal cultivation to unleash renewable fuel productivity
Source: Nat Commun. 2022 Jan 27;13:541. doi: 10.1038/s41467-021-27665-y (PMC8795378; doi:10.1038/s41467-021-27665-y)
Supplement: Supplementary file 1 — Supplementary information [file 41467_2021_27665_MOESM1_ESM.pdf]

Machine learning-informed and synthetic biology-enabled semi-continuous algal cultivation to unleash renewable fuel productivity

Long *et al.*

Supplementary Method 1. Training data collection and processing for light pattern prediction

In order to access real-time light availability inside algal culture, we first developed a light distribution pattern prediction model (LDPM) to predict light distribution patterns (LDPs) in a cuboid photobioreactor (PBR). We collected 138 LDPs in the PBR (19.6 cm in length × 9.6 cm in width × 20 cm in height) with 23 different cell concentrations and 6 different light intensities as training samples for the machine-learning model. The LDPs were captured by a camera fixed on top of a PBR containing different cell concentrations and illuminated with different light intensities. A LED light bar (4000K, CRI =80) placed on one side of the photobioreactor was used as a light source (Figure 1A). The illuminance was monitored by a sensor on the surface of the photobioreactor and converted to photosynthetic photon flux density (PPFD) with a coefficient of 56. The twenty-three cell concentration gradients were set to 0.11973, 0.21294, 0.40872, 0.45162, 0.54405, 0.62712, 0.74256, 0.82056, 0.90948, 0.96915, 1.10604, 1.2246, 1.3026, 1.3923, 1.443, 1.5444, 1.7901, 1.9188, 2.0241, 2.3556, 2.535, 2.9601, 3.6777 g/L, while the six light intensity gradients were set to 107, 178, 267, 357, 570, 714 μmol m^{-2} s^{-1}. The camera was set to manual mode and all parameters were locked throughout the photographing process to ensure consistency. After acquiring all LDPs, raw pictures were cropped, converted to grayscale, and compressed to 40 × 18 pixels in Photoshop 2020 (Figure 1A). The compressed images were used to represent the light distribution pattern inside the photobioreactor with grayscale values (GSVs) representing light intensities. The GSVs were extracted from the grayscale images with the CV2 module in Python. To evaluate the accuracy of GSVs representing light intensities, we extracted GSVs at (0, 20) (row 0, column 20) from

LDPs over a wide range of cell concentrations and assessed the linearity between GSV and light intensity.

The training sample collection for pond LDPM is shown in Figure S10A. LDP images were captured from a simplified pond setup (Figure S10A) and the collected LDP images were converted to 208 × 10-pixel grayscale images as mentioned above. The light intensities for the pond LDP training samples were set to 196, 268, 357, 446, 536, 625, 714, 804, 964, 1071, 1161, 1250, 1339, 1429, 1518, 1607, 1696, 1786, 1875 μmol m^{-2} s^{-1}, and cell concentrations were set to 0.062, 0.140, 0.228, 0.337, 0.466, 0.620, 0.871, 0.999, 1.177, 1.254, 1.396, 1.482, 1.553, 2.007, 2.320, 2.814, 3.199, 3.694, 4.577, 5.519 g/L. The LDP for the pond system was set to be one-dimensional and represented with the 208 pixels in the middle column (column 5) of the LDP image.

Supplementary Method 2. LDPM training and evaluation

Due to the high complexity of the LDP inside algal culture and limited training samples, we believe that predicting LDPs pixel by pixel is the best method for accurate prediction. Pixel-by-pixel prediction means that individual pixels in LDP images are treated as individual models and then combined, rather than treating the whole image as one model. Thus, we trained 720 models for a 40 × 18-pixel LDP prediction. Cell concentrations and light intensities, two major factors shaping LDP, were set as features in training with the corresponding GSVs at each pixel as labels. Both features and labels were normalized by subtracting their average and dividing by their standard deviation. Around 10% of the training samples were randomly selected as testing samples. We chose Support Vector Regression with a Radial Basis Function kernel (SVR-RBF) as the algorithm and kernel for the prediction in this study. SVR-RBF from an open-source machine learning library, scikit-learn[1], was used for training and prediction. We selected the best models at each pixel by selecting the combinations of parameters (C:1, 10, 100, 1000, 3000; gamma: 0.003, 0.01, 0.03, 0.1, 0.3,1.) returning the highest R^2 score. Prediction accuracy was determined by overall evaluation and by pixel-by-pixel evaluation. The overall evaluation calculated an R^2 value by comparing all predicted GSVs with measured GSVs in the testing data set to assess the overall prediction accuracy of the model. Pixel-by-pixel evaluation calculated the R^2 value at each pixel to assess the prediction accuracy at different positions on LDPs. Accuracy percentages were calculated by counting pixels with an R^2 score larger than 0.90 (or between 0.79 and 0.85), and dividing by 720. The R^2 evaluation was performed with the metrics module on scikit-learn. The matplotlib module in Python was used for visualization of evaluation results and predicted images[2].

Supplementary Method 3. Dark area calculation

In the machine-learning training process, we collected LDPs from a larger cuboid photobioreactor (19.6 cm in length × 9.6 cm in width × 20 cm in height) in order to get more information from a single image. However, the photobioreactor used for cultivation was a smaller photobioreactor 10 cm long and 5 cm wide. To adapt the pre-trained models to the smaller photobioreactor, we selected the 10 left-most columns (column 0-9) and 10 right-most columns (column 30-39) in the 9 rows (row 0-8) closest to the light source in the LDP of the larger photobioreactor to represent the LDP of the smaller photobioreactor. Thus, LDPs in small photobioreactors were represented by images with 180 (20 × 9) pixels. For dark area calculation, grayscale values less than 25.5 (1/10 of the maximum grayscale value) were counted (n) and normalized as percentages of LDP pixels (n/180×100%). The dark area with double light sources was estimated with the following equation (1), assuming no interference between light from two sources:

$$A_2 = \begin{cases} (1-(1-A_1)\times 2)\times 100\% & if\ A_1 > 50\%; \\ 0 & if\ A_1 < 50\%; \end{cases} \quad \textbf{(1)}$$

Where A_1 and A_2 refer to dark areas with one and two light sources at given light intensities, respectively.

Supplementary Method 4. Growth curve fitting, growth rate calculation, and biomass productivity prediction

To generate a growth curve, we collected cell concentration under given light intensities at different time points by measuring OD_{730}. Variables were normalized by subtracting their average and dividing by their standard deviation. The logistic curve was defined as the equation (2):

$$f(x) = \frac{a}{(1 + e^{-c(x-d)})} + b \qquad \textbf{(2)}$$

Where *x* represents the variable here, representing time and *a, b, c, d* are parameters that determine the shape of the growth curve. The fitting and prediction were processed by the Optimize module in the SciPy library in Python[3]. Growth rates at specific time points were estimated by the slope of the corresponding curve at that point.

Supplementary Method 5. Growth Rate Prediction Model (GRM) training and evaluation

The GRM was trained to predict cyanobacterial growth rates based on the LDPs (Figure 2B). In order to collect training data, we cultivated cyanobacteria under different light intensities (107, 178, 267, 357, 570, 714 μmol m^{-2} s^{-1}) in the smaller PBR. The concentration of the cyanobacteria was monitored and fitted with sigmoid curves for growth rate calculations mentioned above. Vectors extracted from the first 9 rows in the middle column of the LDP were used as features, and the corresponding (at the same time points) calculated growth rates were set as labels in the training. The features were normalized by subtracting their averages and dividing by their standard deviations. The random forest algorithm was used for the GRM model and the performance of the model was evaluated by calculating an R^2 value between the predicted and measured growth rates in the reserved testing set (20% of the training samples). The GRM was adapted to predict growth rates under a double-light condition based on the assumption that there are no interferences between light from two sources. In this way, vectors extracted from the first 5 rows of the middle columns of LDPs were used as features for the GRM training.

Similar to the GRM for PBR, we grew several batches of cyanobacteria in a pond system to acquire the growth data for pond GRM training. The growth data were then fitted with sigmoid curves for growth rate calculation. The normalized 208-length vectors predicted from the pond LDPM and the calculated growth rates were set as features and labels, respectively, for the pond GRM training. The training and evaluation of the pond GRM were the same as the PBR GRM described above.

Supplementary Method 6. Growth simulation

Cyanobacterial growth simulation was performed as shown in Figure 2A. An initial cell concentration and light program are required inputs and the simulation process contains a loop with four steps: 1) predict the LDP based on the initial cell concentration and initial light intensity with the LDPM; 2) predict the growth rate based on the LDP from step 1 with the GRM; 3) calculate the new cell concentration from the initial cell concentration and the predicted growth rate; 4) update the newly calculated cell concentration and current light intensity as inputs for the next round of LDP prediction. The light programs were specified in the main text. The initial cell concentration used for PBR growth simulation ranged from 0.2 to 4.8, with a 0.2 increment. The initial concentration used for pond growth simulation was set to 0.1, 0.4, 0.6, and 0.8. To ensure accurate growth simulation, the bubbling rate, temperature, surface area, and light conditions were tightly controlled in a way that no severe sedimentation happens during cultivation in PBRs, while these conditions were controlled to achieve sedimentation in collection vessel.

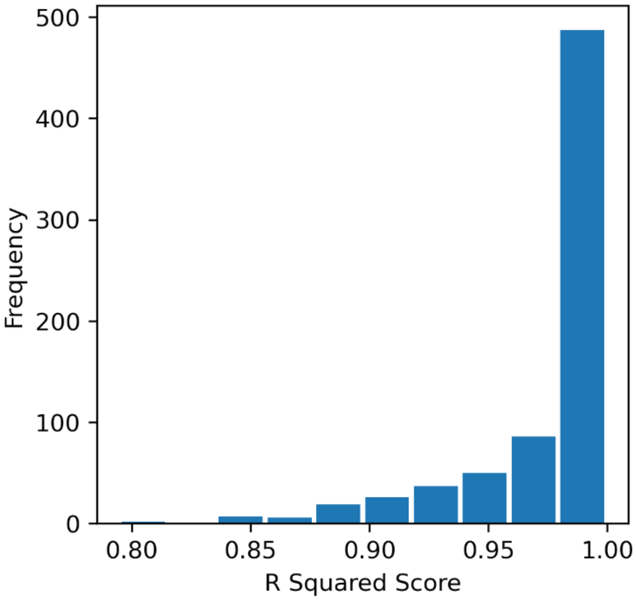

Supplementary Figure 1. Pixel-by-pixel evaluation of LDPM prediction over testing samples. 94.4% of pixels achieved R^2 values higher than 0.90, and only 0.8% of pixels had R^2 values in the range of 0.79 to 0.85.

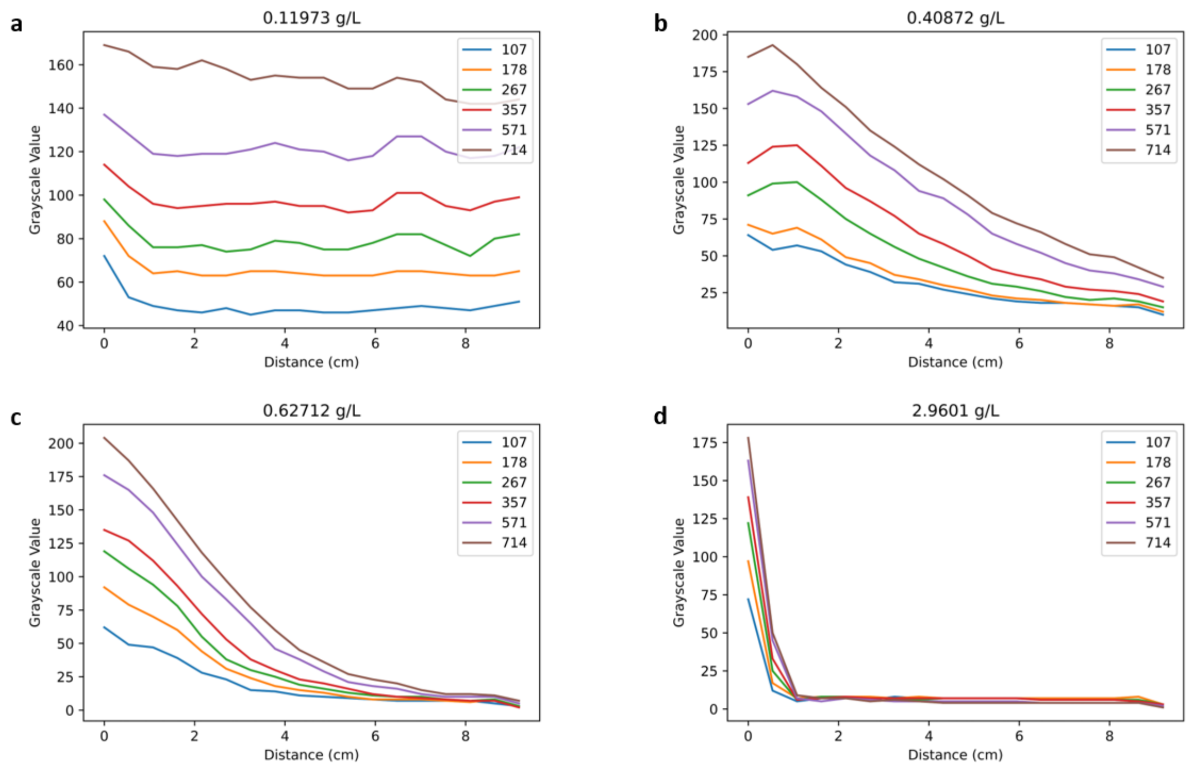

Supplementary Figure 2. Light intensity (represented by GSV) changes over the length of the light path. GSVs in the middle column (column 20, row 1 - 18) of LDPs were extracted to represent light intensities over light paths and plotted against distances from light sources (a-d). Different colors in the figures represent different intensities of light sources (107, 178, 267, 357, 570, 714 μmol m^{-2} s^{-1}). Light intensity decreased only slightly over the path when cell concentration was low (a). But significant decreases were observed when cell concentration increased (b, c), and light intensity dropped sharply (GSV below 20 within 1 cm) when cell concentration reached 2.9601 g/L (d). The results suggest intensified mutual shading at higher cell concentration. Source data are provided as a Source Data file.

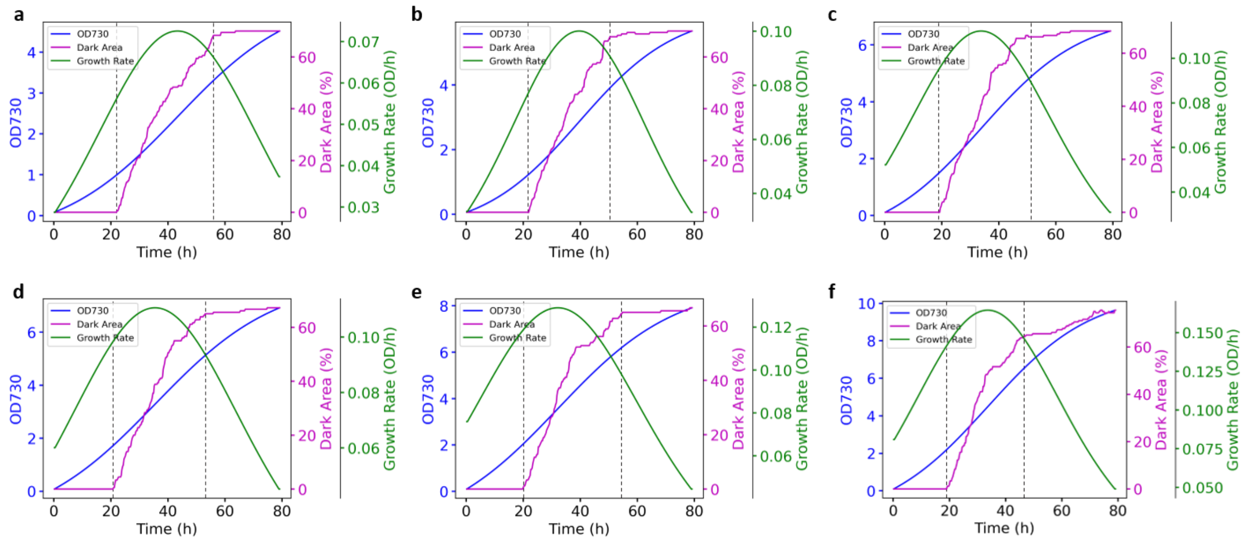

Supplementary Figure 3. Relationship between cell concentration (OD_{730}), dark area derived from LDP, and growth rate. Growth curves (blue) were generated by fitting cell concentration (OD_{730} and time) collected from cultivations under light intensities at 107 (a), 178 (b), 267 (c), 357 (d), 570 (e), 714 (f) µmol m^{-2} s^{-1}. Slopes of growth curves were calculated to represent growth rates at these light intensities (green, a-f). LDPs over the growth at given light conditions were predicted by LDPM and dark areas were defined as LDP pixels with GSVs less than 25.5 (magenta, a-f). Growth rates peaked at 36.8 ± 4.7 hours and dark areas reached 43.1 ± 4.9% regardless of light intensity (green, magenta, a-f). Overall, dark areas experienced three stages: zeros stage (left of the first dashed line), increasing stage (in between the first and second dashed lines), and plateau stage (right of the second dashed line) (magenta, a-f). The increasing stage (between dashed lines) overlapped significantly with the fastest growth period of cyanobacteria (green, magenta, a-f). Source data are provided as a Source Data file.

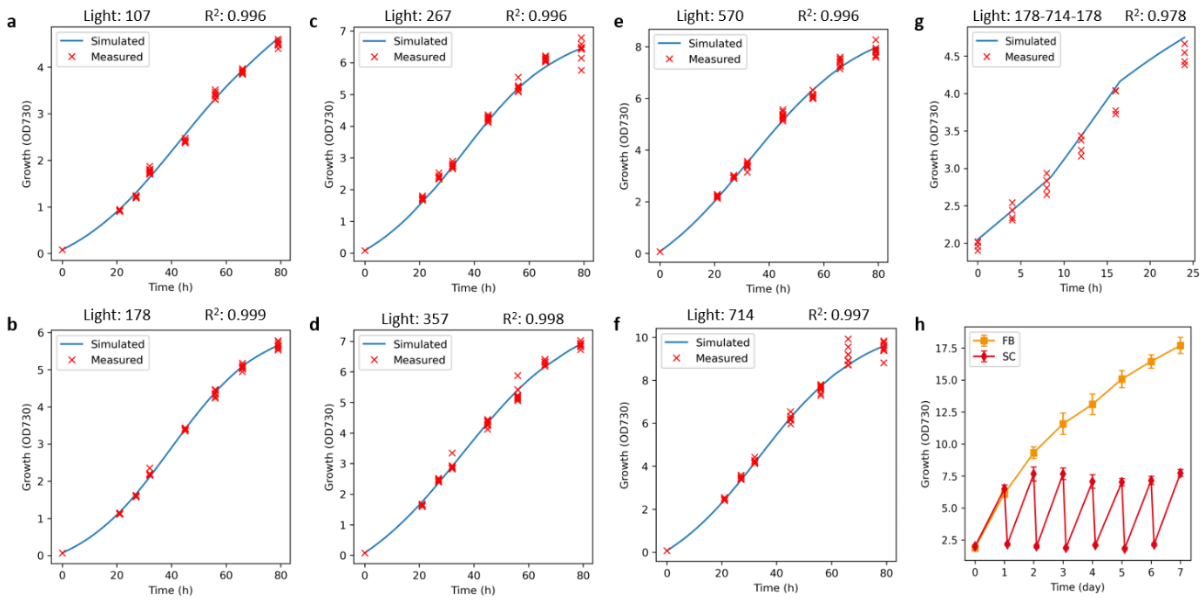

Supplementary Figure 4. Validation of growth simulation by machine-learning models under different growth conditions and comparison between semi-continuous algal cultivation (SAC) and fed-batch. The growth simulation achieved R^2 scores of 0.996 (a), 0.999 (b), 0.996 (c), 0.998 (d), 0.996 (e), 0.997 (f), and 0.978 (g) under light intensities of 107, 178, 267, 357, 570, 714, and 178-714-178 µmol m^{-2} s^{-1}, indicating high prediction accuracy. (h) Growth comparison of UTEX 2973 with SAC and fed-batch cultivation Data are presented as mean values +/- standard deviations (n = 3 independent samples). Source data are provided as a Source Data file.

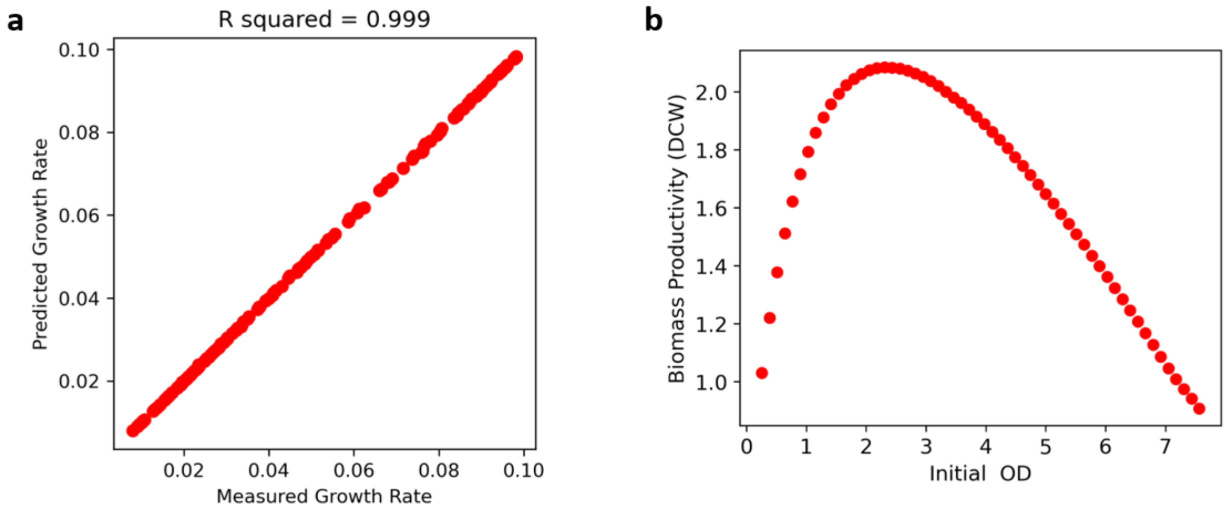

Supplementary Figure 5. GRM adapted for growth rate prediction with double light sources. a, validation of the growth rate prediction by the GRM adapted for double light. b, Growth simulation suggests setting initial OD_{730} at 2.3 delivers highest biomass productivity. Source data are provided as a Source Data file.

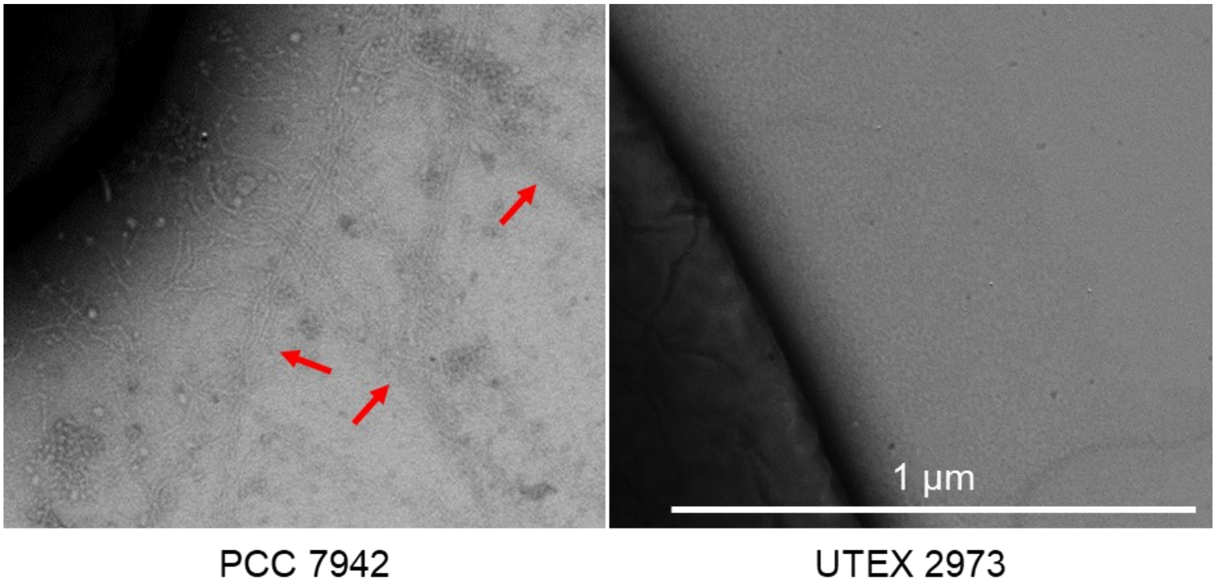

Supplementary Figure 6. Transmission electron microscopy reveals cell surface differences between *Synechococcus elongatus* PCC 7942 and UTEX 2973. UTEX 2973 showed relatively smooth cell surface compared to PCC 7942, where lots of pili formed. Similar results were found in two independents observations. Original images are provided as a Source Data File.

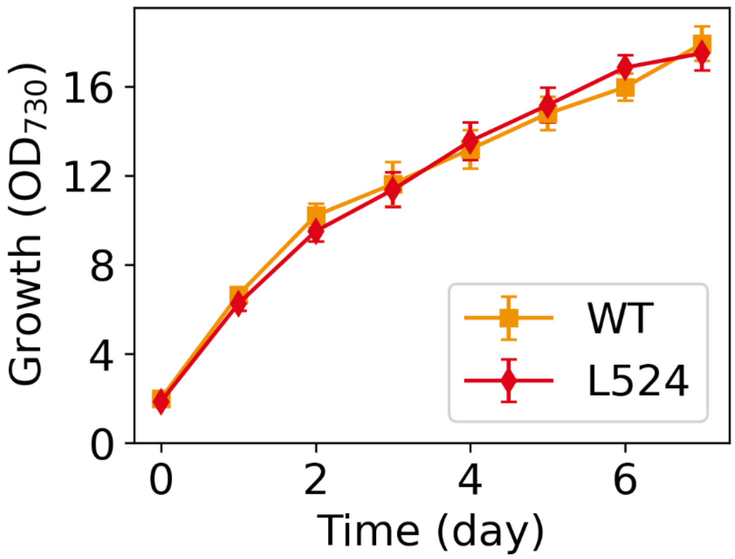

Supplementary Figure 7. Comparison of growth between UTEX 2973 WT and L524. No significant growth differences were found between WT and L524 in the given growth conditions. Data are presented as mean values +/- standard deviations (n = 3 independent samples). Source data are provided as a Source Data file.

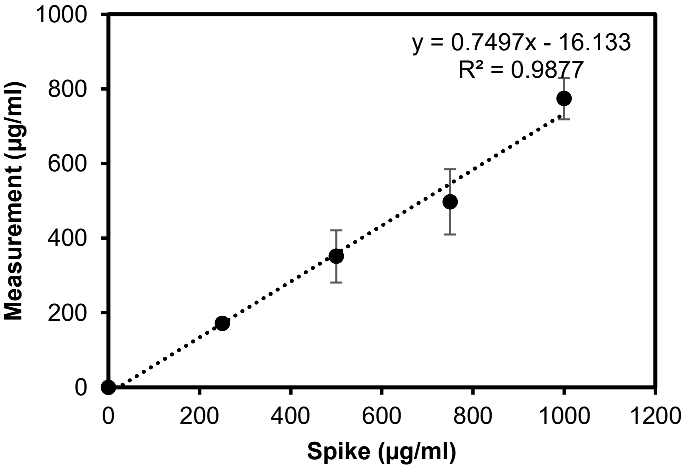

Supplementary Figure 8. Standard curve used to normalize limonene productivity with recovery rate. Limonene concentrations of 250, 500, 750, 1000 µg/mL were used to spike the UTEX 2973 wildtype cells. Limonene was collected and measure as described in the Methods of the main text. Data are presented as mean values +/- standard deviations (n = 3 independent samples). Source data are provided as a Source Data file.

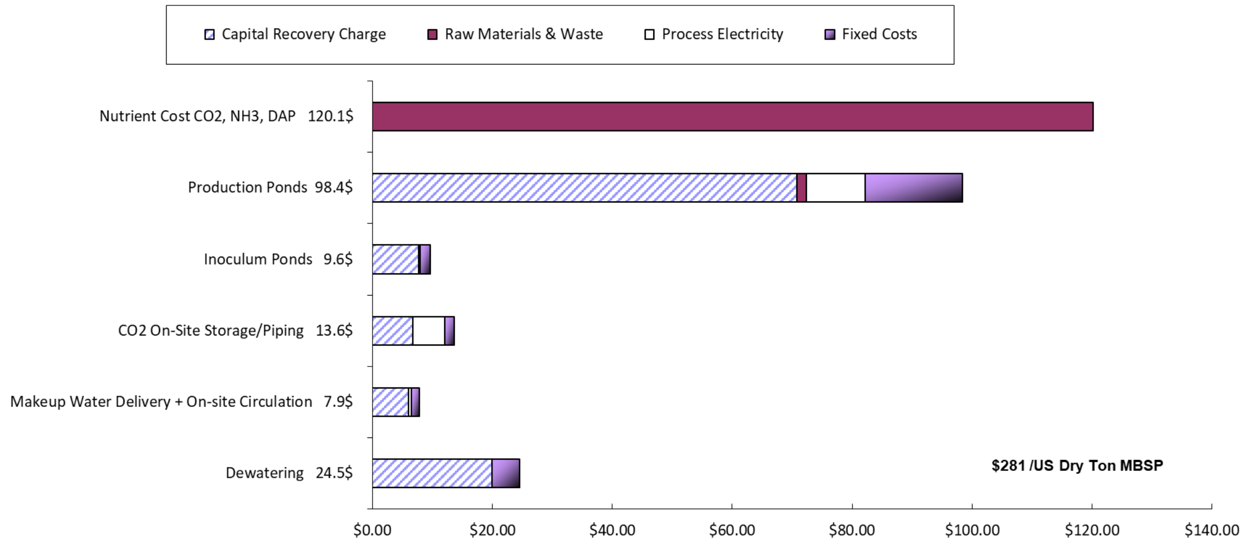

Supplementary Figure 9. Techno-economic analysis of the pond SAC platform. The NREL algae farm model projects a MBSP of approximately $281 per ton based on the outdoor trial yield. Cost breakdown suggests the dewatering process accounts for $24.50 per ton.

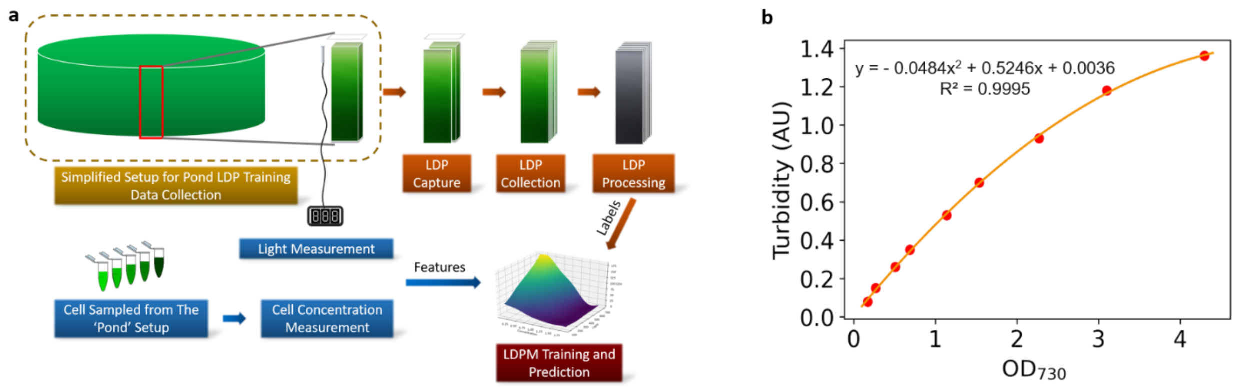

Supplementary Figure 10. LDP prediction for open pond system and conversion between turbidity and OD_{730}. The process of adapting LDPM for pond system prediction (a) and the calibration curve for OD-turbidity (Attenuation Unit, AU) conversion (b). Source data are provided as a Source Data file.

Supplementary Table 1. Primers used in this study

Primer name	Sequence	Note
NS-DS-F	cacgaggccctttcgtcttcaagaaATGGATCTGACCAACATG	Building L524
NS-US-R	atcgatgataagctgtcaaacatgagaaAAACGCGCGAGGCAGGAT	Building L524
NSI-F	TCAGCTGCTTTAGGCCCACCAGTTTGAAG	Segregation
NSI-R	TTATCTCTCGGCTAGTGGACGCAAGCAGCG	Segregation
petB1-F	CGACTGGTTCGAGGAGCGTC	qRT-PCR, IS
petB1-R	TTGCAAAGCCGGTGGCAAAC	qRT-PCR, IS
LS1-F	CTCGAATCTGCCCGCGAGTT	qRT-PCR, LS
LS1-R	GATCCAGACCGGGGCATTGG	qRT-PCR, LS

IS, internal standard; LS, limonene synthase.

Supplementary references

1. Pedregosa F*, et al.* Scikit-learn: Machine Learning in Python. *J Mach Learn Res* **12**, 2825-2830 (2011).

2. Barrett P, Hunter J, Miller JT, Hsu JC, Greenfield P. matplotlib - A portable python plotting package. *Astr Soc P* **347**, 91-95 (2005).

3. Virtanen P*, et al.* SciPy 1.0: fundamental algorithms for scientific computing in Python. *Nat Methods* **17**, 261-272 (2020).
