# Supplementary material for: Machine learning-informed and synthetic biology-enabled semi-continuous algal cultivation to unleash renewable fuel productivity
Source: Nat Commun. 2022 Jan 27;13:541. doi: 10.1038/s41467-021-27665-y (PMC8795378; doi:10.1038/s41467-021-27665-y)
Supplement: Supplementary file 3 — Description of Additional Supplementary Files [file 41467_2021_27665_MOESM3_ESM.pdf]

Description of Additional Supplementary Files

File Name: Supplementary Movie 1
Description: Video showing the aggregation-based sedimentation in Eppendorf tubes. The video was speeded up for 32 times and recorded the first seven minutes of the sedimentation.
