# Supplementary material for: Machine learning-informed and synthetic biology-enabled semi-continuous algal cultivation to unleash renewable fuel productivity
Source: Nat Commun. 2022 Jan 27;13:541. doi: 10.1038/s41467-021-27665-y (PMC8795378; doi:10.1038/s41467-021-27665-y)
Supplement: Supplementary file 5 — Reporting Summary [file 41467_2021_27665_MOESM5_ESM.pdf]

nature portfolio

Corresponding author(s): Joshua Yuan

Last updated by author(s): Oct 20, 2021

Reporting Summary

Nature Portfolio wishes to improve the reproducibility of the work that we publish. This form provides structure for consistency and transparency in reporting. For further information on Nature Portfolio policies, see our Editorial Policies and the Editorial Policy Checklist.

Statistics

For all statistical analyses, confirm that the following items are present in the figure legend, table legend, main text, or Methods section.

n/a	Confirmed	
☐	☒	The exact sample size (*n*) for each experimental group/condition, given as a discrete number and unit of measurement
☐	☒	A statement on whether measurements were taken from distinct samples or whether the same sample was measured repeatedly
☐	☒	The statistical test(s) used AND whether they are one- or two-sided<br>*Only common tests should be described solely by name; describe more complex techniques in the Methods section.*
☒	☐	A description of all covariates tested
☒	☐	A description of any assumptions or corrections, such as tests of normality and adjustment for multiple comparisons
☐	☒	A full description of the statistical parameters including central tendency (e.g. means) or other basic estimates (e.g. regression coefficient) AND variation (e.g. standard deviation) or associated estimates of uncertainty (e.g. confidence intervals)
☐	☒	For null hypothesis testing, the test statistic (e.g. *F*, *t*, *r*) with confidence intervals, effect sizes, degrees of freedom and *P* value noted<br>*Give P values as exact values whenever suitable.*
☒	☐	For Bayesian analysis, information on the choice of priors and Markov chain Monte Carlo settings
☒	☐	For hierarchical and complex designs, identification of the appropriate level for tests and full reporting of outcomes
☒	☐	Estimates of effect sizes (e.g. Cohen's *d*, Pearson's *r*), indicating how they were calculated

Our web collection on statistics for biologists contains articles on many of the points above.

Software and code

Policy information about availability of computer code

Data collection	Vernier Graphical Analysis was used for light monitoring and EXpert software was used to measure the growth in the pond system.
Data analysis	Customized codes was used to perform the machine learning. The codes were writen in Python3 (3.8.2) and based on scikit-learn (0.23.1) and implemented with jupyter notebook (6.0.3). Packages including numpy (1.18.2), matplotlib (3.2.1), cv2 (4.2.0), scipy (1.4.1), and pandas (1.0.3) were used to implement the machine learning and visualization. All codes as well as training data will be available from https://github.com/joshuayuanlab151/LDPM-and-GRM/

For manuscripts utilizing custom algorithms or software that are central to the research but not yet described in published literature, software must be made available to editors and reviewers. We strongly encourage code deposition in a community repository (e.g. GitHub). See the Nature Portfolio guidelines for submitting code & software for further information.

Data

Policy information about availability of data

All manuscripts must include a data availability statement. This statement should provide the following information, where applicable:

- Accession codes, unique identifiers, or web links for publicly available datasets
- A description of any restrictions on data availability
- For clinical datasets or third party data, please ensure that the statement adheres to our policy

The experimental data that support the findings of this study are available as a supplement to this manuscript (Supplementary figures and tables). Training data for machine learning models are available from GitHub. Source data are provided with this paper.

Field-specific reporting

Please select the one below that is the best fit for your research. If you are not sure, read the appropriate sections before making your selection.

- [x] Life sciences
- [ ] Behavioural & social sciences
- [ ] Ecological, evolutionary & environmental sciences

For a reference copy of the document with all sections, see nature.com/documents/nr-reporting-summary-flat.pdf

Life sciences study design

All studies must disclose on these points even when the disclosure is negative.

Sample size	During developing machine learning models for the Photobioreactor prediction, 138 LDP images with 23 gradients of cell concentrations and 6 gradients of light intensities were sampled for LDPM training. The sample size is reasonably large enough as the model shows high prediction accuracy on testing samples (average R-squared value: 0.995). 114 samples were collected for GRM training. The high prediction accuracy (R-squared value: 0.992) on testing samples suggests that the sample size is large enough. During developing machine learning for the pond system, 380 LDP images with 20 gradients of cell concentrations and 19 gradients of light intensities were sampled for LDPM training. The high accuracy of pond LDPM (r2 = 0.986) and GRM (r2 = 0.980) suggested that the sample size is large enough for precise predictions. Three biological replicates with 2-3 technical replicates were set in the PBR cultivation , limonene testing, BATH assay, and sedimentation testing. The similar results from each replicates suggested the sample is sufficient for the experiments. Due to the throughput limitation, 2 biological replicates were set for the indoor and outdoor pond cultivations. The consistence of results from these experiments suggested that the sample size is large enough. No statistical methods were used to determine the sample size.
Data exclusions	No data were excluded from analysis.
Replication	2-3 biological replicates and 2-3 technical replicates were set for measurements in the study. All replicates show similar results.
Randomization	During machine learning training, the training samples and testing samples were selected randomly using random module in Python. Randomization is not applicable for other experiments presented in this study, as the experiments like strain engineering, cultivation design, and sedimentation testing are rationally designed, and the results are cross-validated by replicates. No statistic analysis was performed in order to reach the main conclusion of the study.
Blinding	During cyanobacterium cultivation and related measurements, order number, instead of sample/treatment names were labeled. No blinding is necessary for other experiments because the differences from different strains, cultivation designs were very obvious, and the results were further supported by independent biological replicates.

Reporting for specific materials, systems and methods

We require information from authors about some types of materials, experimental systems and methods used in many studies. Here, indicate whether each material, system or method listed is relevant to your study. If you are not sure if a list item applies to your research, read the appropriate section before selecting a response.

Materials & experimental systems

n/a	Involved in the study
☒	☐ Antibodies
☒	☐ Eukaryotic cell lines
☒	☐ Palaeontology and archaeology
☒	☐ Animals and other organisms
☒	☐ Human research participants
☒	☐ Clinical data
☒	☐ Dual use research of concern

Methods

n/a	Involved in the study
☒	☐ ChIP-seq
☒	☐ Flow cytometry
☒	☐ MRI-based neuroimaging
